# Women’s access to sexual and reproductive health services and information in Ismailia, Egypt

**DOI:** 10.1186/s12905-024-02986-4

**Published:** 2024-03-06

**Authors:** Reem Elsayed, Wanga Zembe-Mkabile

**Affiliations:** 1 Researcher and Technical Officer, Egypt Healthcare Authority, 7th Al Nasr Road, Elforsan Building, Nasr City, Cairo Egypt; 2 Senior Specialist Scientist, Health Systems Research Unit, South African Medical Research Council, Cape Town, South Africa

**Keywords:** Sexual health, Reproductive health, Married women, Sexual and reproductive health services, Experiences

## Abstract

**Background:**

Sexual and reproductive health (SRH) is a right that should be guaranteed to every woman worldwide in order to have a healthy and safe sex life. In most Arab countries, including Egypt, there are different cultural, political, and religious factors that have contributed significantly to how society views and treats women’s bodies and sexuality. As a result, it is difficult to provide solid data and information to guide policymakers, policies, and to implement awareness and preventive programmes. This study sought to address this gap by looking at the intersectionality of women’s access to SRH services and information in Ismailia, Egypt.

**Methods:**

The study utilised qualitative research methods. Semi-structured interviews were conducted with twelve married women and two key informant interviews with health professionals (a gynecologist and a pharmacist) in the study area.

**Results:**

The study revealed that married women suffer from scarcity of understanding and knowledge of their SRH and lack of access to adequate SRH services and information. Married women’s experiences of accessing SRH services and information were influenced by intersecting factors located at the micro and macro levels. These intersected factors (e.g., power dynamics, socioeconomic factors, cultural norms, and religious misconception) shaped oppression and privilege structures which created unequal access to SRH information and services.

**Conclusion:**

There is a need for building quality parental relationships for women before and after marriage in order to promote positive SRH attitudes and behavior. There is an urgent need to empower women before and after marriage with accurate, safe, and affordable SRH services and information that could have life-long benefits to protect them. There is a need to conduct educational programmes, and initiate media awareness campaigns, to equip women with information and knowledge about their SRH services and information.

## Background

According to the World Health Organization [1], the sexual health of women is defined as a physical, mental, and emotional state of being, as well as social well-being related to sexuality. Sexual health demands a positive and deferential approach towards sexual relationships and sexuality. It also indicates the potential of an individual having a pleasant and safe sexual relationship that is free of being violated or discriminated against. There are different factors that influence women’s sexual health, such as physical, psychological, interpersonal and social factors (WHO, 2006). Sexuality is an essential element of being a human being, which includes sex, gender identities, sexual orientation, pleasure, and intimacy. It is also affected by different aspects such as physical, hormonal, psychological, economic, social, cultural, religious, legal, political and ethical issues [1–3]. Reproductive health (RH), which is part of sexual health, can be defined as the ability to have a desirable and safe sexual life with the capability to reproduce and having the freedom to determine if, when, and how to do so. Reproductive health also refers to the right to access proper health care services that enable women to have a safe pregnancy and delivery, and that give parents better chances to have a healthy child [1]. Reproductive health has been included as part of sexual health and refers to the provision of appropriate care and consultation in relation to reproduction and sexually transmitted diseases [1]. In 2015, The Sustainable Development Goals (SDGs), in particular, Target 3.7 declared that “Sexual and reproductive health: By 2030, ensure universal access to sexual and reproductive health-care services, including for family planning, information & education, & the integration of reproductive health into national strategies.”

According to WHO (2008), the International Conference for Population and Development (ICPD) in 1994 acknowledged that “universal access to reproductive health” is a development goal that should be accomplished. After that, the World Health Assembly, held in 2004 assured that the international community would show commitment and support to promote SRH and rights through adopting global health strategies to achieve the development goals [4]. The World Health Assembly in 2004 reassured the international community committed to promoting SRH through fostering RH strategies globally in order to achieve the development goals [4, 5].

Most Arab countries including Algeria, Bahrain, Djibouti, Kuwait, Jordan, Libya, Morocco, Oman, Qatar, Saudi Arabia, Palestine, Sudan, Syria and Lebanon address SRH in accordance with religious values and societal traditions. These countries provide basic SRH services such as family planning and administer contraceptives [6].

However, deeply rooted values and traditions play a vital role in formulating and shaping the identity and the mindset of Arab society [7, 8]. For example, Stephan (2006) argues that religion plays a crucial role in shaping the mindset of Arab society as it relates to women’s bodies, their sexuality and SRH. She indicated that both Muslims and Christians in the Middle East have drawn on traditions and beliefs to justify violence and oppression against women [9]. Stephan (2006) and Sensenig (2002) further state that both men and women have created their own interpretation of what religious values are regarding women’s bodies and sexuality and the cultural framework has significantly contributed to the way society views women’s sexuality and the value of their virginity and honour. These authors further note that Arab states and the family have implicit consent on women’s sexuality through dictating the amount of SRH information and services that they should know or receive. They acknowledged that misinterpretation of religion and the patriarchal foundations in Arab society makes it difficult to achieve significant change that promotes women’s rights and addresses gender-based violence [9, 10]. El-Kak, (2013) and Obermeyer (2015) concurred that the huge value that the Arab society places on the virginity of unmarried girls and their sexuality plays a crucial role in subjecting them to extreme social stigma, judgment and discrimination by health practitioners if they attempt to obtain contraceptives or seek information regarding sexuality and sexual health. As a result, young married and unmarried women suffer from a lack of access to SRH knowledge and contraceptive services [11, 12].

In this regard, Shaw (2018) states that the understanding of sexual health in Egypt is clouded by misconceptions and strong cultural stigma that jeopardizes women’s sexual health and discourages them from seeking medical care. It is for this reason that this research intends to address some of these gaps to provide insight and understanding into the reasons and consequences of the lack of women’s accessibility to SRH services and information [13]. The SRH services that are provided in the study area are RH and family planning, pregnancy follow-up, safe delivery, early detection of tumours, education for breastfeeding, early detection, and referral of high-risk pregnancies [13].

The Regional Conference on Population and Development, “Development Challenges and Population Dynamics in a Changing Arab World”, which took place in Cairo in June in 2013, acknowledged that the advancement and protection of SRH rights are not only fundamental to the recognition of social justice and assuring a healthy and secure life but are also fundamental to achieving national and global sustainable development goals [14]. As such, there is a recognizable need for the allocation of appropriate resources and funds to ensure access to quality SRH services [15, 16].

The topic of sexual health is sensitive and controversial in Egypt as it is considered one of the main taboos in the country. Mohamed Mustafa (Shaw, 2018), a lawyer of a feminist organization in Egypt, states that the different violent sexual practices against women such as sexual harassment and domestic violence in Egypt serve a political purpose in that it discourages women from political participation [13, 17]. The state thus dictates the quality and quantity of women’s SRH services and information which is used as a mechanism to intimidate and dominate the power dynamics between men and women [13, 17]. This approach is used to maintain male privilege and its rootedness in the male-dominated public sphere of Egyptian society [13, 17, 18].

In Egypt, sexuality and SRH care are under-researched and there is limited data and information on these topics. Further, there are taboos surrounding public discussions about women’s sexuality and SRH and topics related to them such as STIs, female dysfunction and the psychological effects of different violent practices. These issues play a crucial role in shaping and influencing women’s sexuality as well as their sexual health [11, 19, 20]. The understanding of sexual health in Egypt is clouded by misconceptions and strong cultural stigma that jeopardizes women’s sexual health and discourages them from seeking medical care. There are several cultural, religious and political challenges that hinder women’s access to SRH in Egypt. SRH and the related issues such as early marriage, female genital mutilation (FGM), and sexual transmitted infections (STIs) are still considered cultural taboos. Women still suffer from a culture of ignorance and shame for seeking such services, which globally are considered basic rights for every female to have a healthy and safe sex life [1, 13, 17, 21, 22]. Furthermore, religion plays a significant role in forming the mentality of Egyptians with regard to women’s bodies and their sexuality, with both being monitored and policed by the state and society [9]. For instance, in some countries and societies both Muslims and Christians endorse and sanction certain beliefs and conventions that encourage the practice of justified violence and oppression against women, including their SRH [9].

It is for this reason that this article addresses some of these gaps to provide insights and understanding into the reasons and consequences of the lack of women’s accessibility to SRH services and information.

## Study setting

**Fig. 1:**
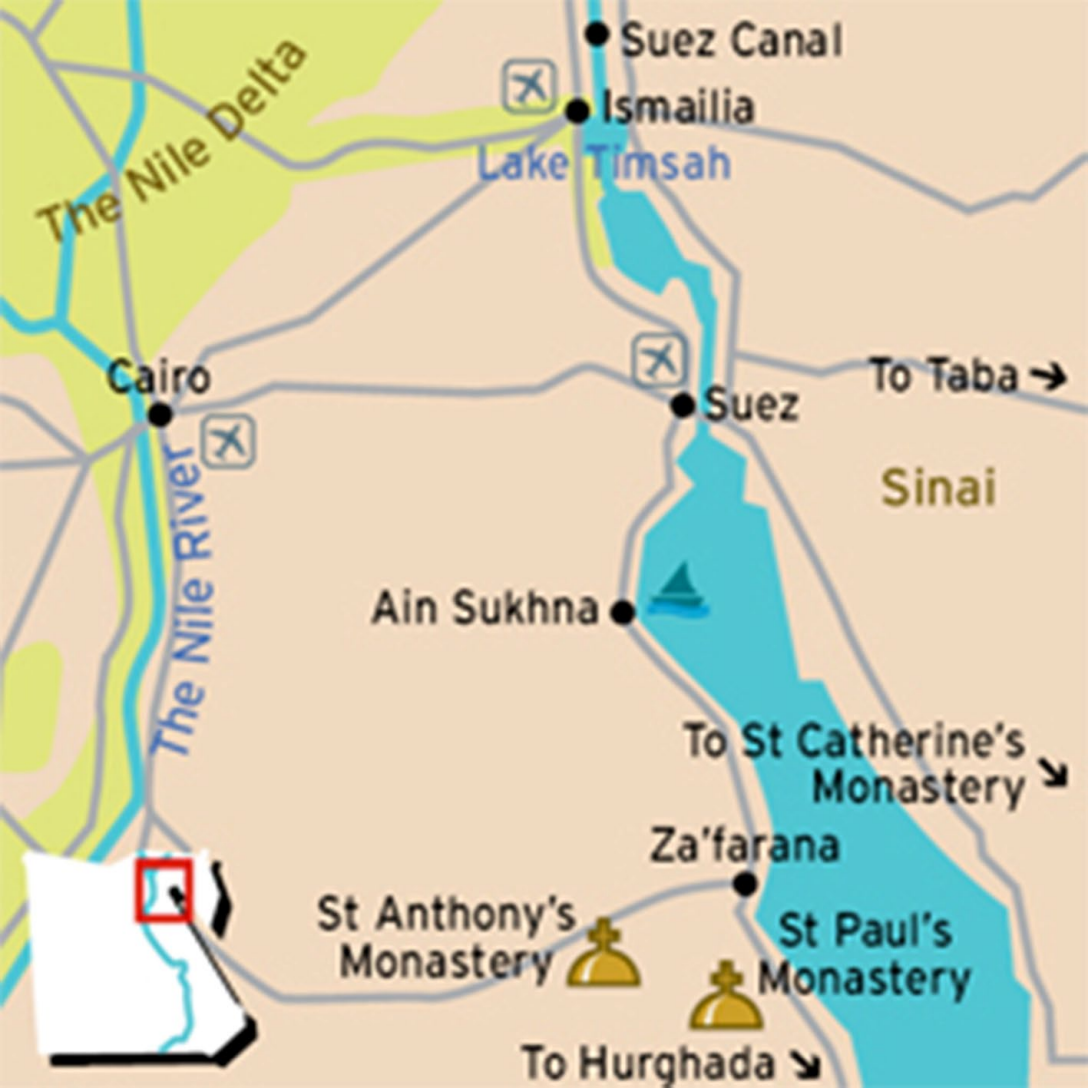
The City of Ismailia in Egypt [25]

The study area is the city of Ismailia, which is located on the west bank of the Suez Canal region in North Eastern Egypt (see Fig. 1) [23]. The Suez Canal connects the Mediterranean Sea with the Indian Ocean and Red sea and was opened in 1869. The city of Ismailia is located 115 km from the capital city, Cairo. The city was founded during the construction of the Suez Canal in 1863 [24].

Egypt comprises of 27 governorates, which are constituted for purposes of administration. A governor, selected by the President, administers each governorate. The Ismailia governorate is one of the Canal Zone governorates and its capital is the city of Ismailia [26]. The Canal region includes three governorates, Port Said, Ismailia, and Suez. The three were built in the 19th century during the construction of the Suez Canal. Port Said is located in the northern entrance of the Canal, and Suez lies at the canal southern end [27]. The city of Ismailia has a total population of 1,432,323, limited job opportunities, and 24.1% of the population lived below the poverty line in 2015 [28].

### Methods

This qualitative study focused on an in-depth examination of the intersectionality of women’s access to (SRH) services and information in Ismailia, Egypt. This study used a qualitative methodology approach because it is considered the most suitable method to collect the required information to achieve the study’s purpose as this research is a very sensitive topic in Egypt. Semi-structured interviews were conducted with fourteen participants, including, twelve married women and two healthcare professionals (a gynecologist and a pharmacist).

The interview guideline was based on a set of questions that specifically addressed the issues related to married women’s access to SRH services and information. Ismailia was purposively sampled as the study area because there is insufficient data about women’s access to SRH services and information. In addition, the findings of the study help to highlight the gaps in women’s access to SRH services and information. The lead author, RE, who is a qualified pharmacist and conducted this research for her master’s degree in Development Studies, and the second author WZ is a senior specialist scientist at South African Medical Research Council in Cape Town, South Africa.

### Sampling methods

This study used purposive sampling and snowball sampling because they were considered the most appropriate sampling techniques for collecting the data that serves the study purpose as well as the nature of the study [29]. Semi-structured interviews were conducted with twelve married women in study area, Ismailia. We used purposive sampling to recruit women who had to be married and sexually active and were therefore meant to access SRH services and information. RE made use of her existing informal networks to access married women. These women referred RE to other married women who are sexually active in order to recruit them through a process of snowball sampling. Snowball sampling was well suited for the study as it focused on a sensitive issue and therefore it was difficult to recruit participants in any other way [30]. This technique relied on referrals, where one participant recruits subsequent others. The snowballing technique was additionally considered to be the appropriate method for the study as it required few cases to assist in encouraging other cases to participate in the study in order to increase the sample size. This approach is most appropriate in small populations that are hard to access because of their closed nature [29]. However, RE kept in mind that the quality of data using snowballing sampling might be biased. This might limit the sample validity because the study participants were based on the subjective choices of the participants first accessed [31]. However, for qualitative research methods it is not necessary to maintain sample validity, as the nature of the methodology does not require generalisation beyond the studied population.

For the key informant interviews, purposive sampling was employed to recruit two health professionals (a gynecologist and a pharmacist) to cover various aspects of the phenomenon from a professional and expert point of view [34]. Purposive sampling was chosen as it selected possible respondents who have experience, knowledge, and perception of the specific phenomenon under study in order to answer the study question [32, 33]. With this sampling method, RE had to put specific criteria for inclusion. Only health professionals who deal specifically with SRH issues, such as male gynaecologists and female pharmacists were approached for inclusion. A male gynaecologist was interviewed as gynaecologists are the main specialists who provide SRH services in Egypt. There are not many female gynaecologists in Ismailia and most practice part-time. The main gynaecologist that was interviewed is very prominent and experienced in the study setting. The lead author, RE met the identified married women and key informants individually. They were given a verbal explanation of the research purpose, how and why they were selected, and what the study process would entail. They were given a participant information sheet with the relevant information as well as a consent form in Arabic. The participants were informed of the date when the signed consent forms would be collected to give them enough time to make an informed decision as to whether they would like to participate in the study. The dates for the interviews were communicated to the participants after making arrangements with them. Each interview took about 45 min to one hour depending on the responsiveness of the interviewee. At the start of each interview, the motivation for conducting the study and the importance of gaining an understanding from their own perspective were explained to each participant again. The importance of being able to share their experiences, suggestions, questions and reservations freely and truthfully to attain information that can assist with improvement of SRH services and information in Ismailia, was explained to the participants After the ninth interview, saturation in the data was reached as no new ideas, themes, patterns or opinions emerged from the data gathered from the participants. There was enough information collected to answer the study questions and further data collection data was not needed [34]. RE conducted all the interviews herself in Arabic, a language that she is fluent in. All interviews were audio-recorded with the permission of the interviewees. In instances where participants did not want to be recorded, RE respected their wishes and relied on notes only.

Table 1 shows the characteristics of the 12 married women with whom semi-structured interviews were conducted. The married women’s ages were between 25 and 55, all of whom had children between the ages of a year and a half to 30. The majority of the interviews had formal education. In regard to their employment status, 10 of the interviewees were government employees and two were unemployed. Two health professionals (a pharmacist and a gynecologist) were also interviewed because of their expertise in providing SRH services and information.

**Table 1:** Demographic characteristics of married women interviewees

Socio-demographic		Frequency	Percentage
Age group	20–30	3	25%
	30–40	5	41.6%
	40–50	1	8.3%
	50–60	3	25%
	**Total**	**12**	**100**
Marital status	Single	0	0
	Married	12	100
	**Total**	**12**	**100**
Educational status	No formal education	0	0
	Secondary education	1	8.3%
	Tertiary education	11	91.6%
	**Total**	**12**	**100**
Employment status	Formally employed	10	83.3%
	Informal employed	0	0
	Unemployed	2	16.6%
	**Total**	**12**	**100**

*Source* Field data

### Data collection and analysis

The lead author conducted a pilot study with three married women in order to refine the study tools to gain more information and reveal more useful questions to use in subsequent interviews. The pilot study helped RE to refine her semi-structured interview schedule. The pilot study was guided by the participants themselves. After refining the tools, they were shared with the co-investigator. Initial codes were put together into categories that were then transformed into major themes. Transcripts were not given back to participants for comments. However, the ethics protocol motivated interviewees to ask questions and the interviewer was careful to reflect back and summarise comments during the interview to ensure the accuracy of the data. The data was presented using descriptions and quotations for illustration. We used qualitative data analysis software, Atlas.ti, which is a useful workbench for analysing qualitative data, particularly visual, text, and audio data. In addition, it offered a comprehensive overview of the research project that is called the Hermeneutic Unit (HU) in Atlas.ti as well as facilitating retrieval functions and immediate search. Atlas.ti also has network-building features that permitted the authors to visually connect chosen memos, texts and codes through means of diagrams [35].

### Patient and public involvement

The development of the study question was informed by past research which indicated women experiencing similar levels of insufficient access to SRH information and services in Egypt. In conducting this study, the authors sought to understand participants’ experiences of accessing SRH information and services. Therefore, the study question speaks to participants’ lived experiences and priorities as it relates to SRH issues that directly affect their well-being.

## Results

Participants were asked to describe in detail their experiences of accessing SRH information and services. The study identified the essential themes and subthemes generated from the field data to capture women’s experiences of accessing to SRH services and information in Egypt. The main theme focuses on the barriers that married women face in accessing comprehensive sexual and reproductive health information and services in Egyptian Society. The sub-themes include gender inequities and societal oppression, and married women’s limited understanding and awareness of sexual and reproductive health.

The study starts off by presenting results related to: (1) How it feels to be a woman living in Egypt; (2) Married women’s understanding of sexual and reproductive health; (3) Women’s access to sexual and reproductive health services and information; and, (4) Married women’s knowledge of the reproductive health services.

### How it feels to be a woman living in Egypt

Most of the women that participated in this study indicated that they felt oppressed, felt unequal to their husbands or male colleagues at work, and struggled to get the same rights that men have in Egyptian society. For example, participant D, a 52 year old Muslim, middle class and educated, reflects on the marginalized status of women’s rights in Egypt, stating that:

*I do not get my rights as a woman. I do feel that women in Egypt are oppressed and do not obtain their rights. They do both women and men’s work. They work like a nurse and a doctor and teacher for their families and a woman for their husbands at night. … The responsibility is too much for women in Egypt. No one helps her and carry that full responsibility. (Participant D).*

Another respondent, participant G highlights the role of gender inequities, patriarchy, and structural factors in the marginal status of women in Egyptian society:

*It feels like you are a subordinate citizen as if both women and men have the same qualifications and skills, and there is an opportunity, men are the ones to be considered because of their sex “male”, despite the fact that the woman could be much better than them. This happens in both work and the household level as well. …um There is certain inequality and injustice because of the patriarchal society where the men are thought of to be in the privilege structure who responsible for everything and can do anything while women are considered wrong all the time even if they are right. (Participant G, 55, middle class, educated, Muslim*).

Participant B (who is 28 year old, middle class, educated, employed, and Muslim) agreed with Participant G’s description of the inequities and injustice that women in Egypt suffer from, stating that: *“[It is] desperate Umm … There are no rights at all. …. such as political, social, and every other right. women are oppressed, no matter their social status.”*

While most respondents criticised the subordinate status of women in Egypt, three women (25% of the sample) stated that they were satisfied and fine with their situation in both their personal and professional life. For example, Participant H, a middle aged, 54 year old university educated woman who works in a government department, states that she does not feel marginalised as a woman:

*It depends on the situation and the people that you are dealing with. I do feel like I am different from other women, I can manage the situation that I am in as I feel like I have my rights or I am trying to get my rights and I do not feel like I am weak. I am a woman because I want to be one. Thank god that I do have the belief and the ability that I can be the woman that I want. That is why I do not allow anyone to oppress me as well as I have a good education and a good job. On the other hand, I do know that the majority of women are oppressed because we live in a patriarchal society.* (Participant H).

Participant H feels that because she has a good education and hence a good job, she is in a better position to exercise her rights or fight for her rights. However, she also acknowledges that – unlike her – most women are oppressed because of patriarchal power relations. By contrast, Participant E, a young (25 years old), lower middle-class woman who is educated but unemployed stated that:

*I am fine. I do not suffer from psychological traumas or complications as well as its very comforting thing in particular in marriage. For example, when I came to conduct the interview, my husband was very understanding. On the other hand, another person could cause trouble.* (Participant E).

### Married women’s understanding of sexual and reproductive health

Most participants did not understand what is meant by SRH. RE had to explain the meaning first in order for them to share their experience in regards to SRH. When Participant B, a young (28), middle class and educated Muslim woman was asked what her understanding of SRH was, she stated that *“According to us, sex is only for making children only [with low voice when mentioning the word “sex”] and only within legal marriage, nothing more.”*

Participant E similarly noted a lack of access to SRH information, saying that *“I do not know anything about SRH as no one talked to me about it.”* (Participant E, lower middle class, 25, educated, unemployed).

In an interesting interview, Participant F, a lower-middle class 39 year old Muslim woman pointedly highlights that the lack of access to SRH information meant that her experience of sexual intercourse was felt in a negative and disgusting manner; however, when she had more SRH information, she experienced sex in a more pleasing and satisfied manner. More specifically, Participant F explains that:

*Before marriage, talking about SRH was indecent and improper. …. As our parents taught us not to talk about [it]and even if they saw us speaking to male neighbours, they beat us. … After marriage. As I married three times, ummm [during] my first marriage, my SRH understanding was terrible, I could not sexually respond to him as I did not know anything about the sexual intercourse and my sexuality …and he also did not tell me anything. … I felt disgusted about the whole sexual intercourse. ……well, I married three times, the first two times, I was really disgusted by sex and sexual intercourse and my previous two husbands said about me that they married a man not a woman …. but the third one made understand my sexual health and sexual wellbeing, and sexual intercourse in a good way so I liked the intercourse and was satisfied sexually with him, that is when I was 36. … [with a sad and bitter voice] I only understood [then] what women’s sexual health means. I really felt so sad for all the past years that I did not understand my sexuality and sexual well-being and that I was disgusted from the sexual relationship that God created in order to please men and women. That is why I got divorced from my first two husbands.* (Participant F, 39, a lower-middle class Muslim).

Participant F’s quote above mentions the importance of access to SRH information and how this opens up possibilities for understanding women’s sexuality and sexual well-being.

Participant G describes her understanding about SRH: *“I only know that I have to maintain my own personal hygiene and my body especially during my period, have good nutri[tion] during pregnancy and delivery.”* (Participant G, Muslim, 55, educated, middle class).

### Women’s access to sexual and reproductive health services and information

The majority of women said that they access information from sources such as social media, TV, friends, and family. Moreover, they only approach a gynaecologist when they face serious health problems. Participant C, a working-class woman, when asked where she got access to SRH services and information, said that she got her information from:

*The internet and I ask my female friends and older people. …As in Egypt, everybody has something to say about everything. For instance, if a woman does not have a background about pregnancy and knows that you are pregnant, she is still going to advise you to take care and do not carry heavy stuff. ….so, you get information from everybody and its similar information, so I do not need to visit a doctor as everybody is doctors themselves.* (Participant C, working class, 38, Muslim, educated, employed).

Participant E, similarly says that she accesses SRH information through research on the internet.

*I only get information from Facebook and friends but I do not take what I read or know from them for granted but I like to search myself for something that is guaranteed…um I get the information from Medical websites but still do not know if such information is right or not.* (Participant E, 25, educated, unemployed, Muslim).

Participant A, an educated middle-aged woman (age 54) highlights the role of culture, in inhibiting discussions of SRH information. Even though she is educated, the cultural taboo of talking about SRH at the household level in Egypt meant that even when her daughter got married, she could not bring herself to enlighten her daughter:

*I did not get any information or services related to my SRH …. Umm, my older married sister only spoke to me about a few things to do, two days before marriage … even my mother was too shy to tell. Even when my daughter got married, I asked one of my female neighbours to tell her as I was too shy myself. As such a topic is too sensitive, there is not a sexual culture in Egyptian society. We have been raised that such [a] topic is taboo, girls were not allowed to sit with married women when they are speaking about sex or SRH.* (Participant A, 54, middle class, educated, Muslim).Women in Egypt get information on SRH through many avenues. For instance, Participant C describes how her husband received information on sexual intercourse through the medium of pornographic movies; however, because this information was not about SRH, she did not know what health complications this had until after the fact. She explains that:

*Before marriage, I did not get any information and my mother was shy to tell me, after marriage …. my husband was telling me what should I do ….um as he has been bold since we got married, he is not shy person…um he gets that information from porn websites and movies and he still watches them till now and sometimes I watch too. I used to practice anal sex with my husband, I enjoyed it at the beginning but I did not know it’s complications until I had anal fraction, bleeding and conceptions and the process of getting rid of the material waste from my body was very painful.* (Participant C, 38, working class, educated, Muslim).

Participant H also stated she got her information from her husband

*Before marriage, I did not know a slightest information about my SRH and no one told me what to do and what not to do. And the only source of information was my husband as he taught me what to do. … He is a man, maybe he got it from movies or read it because his culture and community gave him the space to do whatever he wanted”* (Participant H, Muslim, 54, middle class, educated, employed).

### Married women’s knowledge of the reproductive health services

Married women admitted that they have knowledge of certain RH services such as antenatal care and delivery care but they do not have sufficient awareness or information about family planning, screening and treatment of STDs, that include virus C and HIV. They also had knowledge about various contraceptives methods like contraceptive pills and IUCDs, but without knowing the advantages or disadvantages of using these methods. On the other hand, women did not mention condoms as a method of contraception as it alleged to reduces men’s pleasure during sexual intercourse. Some prefer to ask their friends and use the internet to get that information as Participant’s C and K, respectively, state:

*There is a need to have health professionals, in particular nurses, to treat us humanely and with respect and not mocking us when we ask questions in relation to our SRH …some of us [are] not educated enough and do not have information about the advantage and disadvantages of contraceptives, from IUCDs or pills, as I do not know which proper method to use and I need to learn about birth control and family planning methods.* (Participant C, 38, working class, Muslim, educated employed).

*I do not know enough information about the different contraceptive methods, … the advantages and disadvantages of each of them, and also do not know enough about STDs as I found out that I have Virus C and I did not know that could be transmitted sexually.* (Participant K, 43, middle class, educated, Muslim).

## Discussion

The study aimed to explore the various factors that influence women’s access to SRH services and information in Ismailia, Egypt. Intersectionality theory was used to explain the interactions with different factors such as gender, class, sexuality, geography, age, disability, ability, cultural, socio-economic status, laws, policies, political interests and economic inequalities, religious institutions, and media with the help of the literature review. These interactions take place within interconnected systems and power structures which affect women’s ability to access SRH services and information [36, 37].

The main findings of this study are that the majority of women in the study felt that they were denied their basic SRH rights, and were oppressed and discriminated against based on their gender, and by the power dynamics and structures in a patriarchal country like Egypt. Married women who were interviewed stated that they did not have enough understanding of SRH, that they lacked knowledge about the various types of RH services available to them before marriage, and only accessed basic services after marriage (e.g. from a gynecologist in primary health units and private practices). For instance, they do not know the advantages and disadvantages of each type, as well as the family planning programmes, which reflects on low service utilization. In addition, the poor treatment by health personnel of women and young women when they seek to know more about SRH knowledge and information.

The findings in our study speak to what has been discussed in Rawson and Liamputtong’s (2010) study in Vietnam which stated that young women in that setting were considered to be vulnerable because of their lack of knowledge regarding their SRH. Even though the Vietnamese study focused on young people, there are parallels with the findings of our study which revealed that some of the interviewees got married very young, and hence faced similar challenges to young women in Vietnam [38]. The Vietnamese study further explored important themes that have been found in our study, which indicates that lack of knowledge and understanding about SRH could lead to serious health problems, regardless of age or population group. Cultural factors also play a significant role in how that society perceives sex and sexuality, and the main aspect of young people’s development is the emergence of their sexuality [38]. In the Vietnamese study, the cultural, religious, class, and socio-economic aspects intersected and formed boundaries that influenced the physical and emotional process of young people’s sexual maturation [38].

In a similar vein to our study, Nguyen (2021) argues that sexuality in conservative communities is constructed by the interaction between social structures (e.g. the family) and individuals. The family is considered a major influence in determining the sexual behaviors of young people. The study further stated that due to young people’s lack of understanding and knowledge of their SRH along with their lack of sexual maturation, this could lead them to unsafe sexual practices and consequences such as unplanned pregnancies and sexually transmitted infections (STIs). Such outcomes could adversely affect their health and well-being in adulthood. As such, access to safe, and sufficient sexual health information even during childhood and adolescence is necessary for improved SRH outcomes in adulthood. The women in our study could have benefited from accessing such information when they were younger [39].

Similar to SRH understanding and knowledge discussed in the preceding section, the study findings show that women’s access to SRH services and information in Egypt is sorely lacking. SRH services which are effective but poorly available include freely available sex education programmes and accurate SRH information and services. These findings indicate that there is a lack of sex education programmes among young people in order to maintain the moral code. This moral code condemns premarital sex, preserves the chastity of young women, and denies access to SRH information. In conservative settings such as Egypt and Vietnam, women’s sexuality and the denial of access to SRH information, are strategies that can be perceived as controlling women, their sexuality, and their bodies [39].

[40] In similar settings to our study, Bashour et al. (2015) mentioned that in Sudan and Morocco, SRH services are in general available and accessible to women. Nevertheless, many significant SRH services stay neglected by the public sector. The lack of integration and continuity of care (e.g., poor postnatal care) are persistent problems. The study also claimed that there are inequalities in access to services that are premised on geographical locations (rural and urban) and that wealth/income need to be discussed to promote equity. Generally, the lack of integration, sub-standard quality of free health services, private sector dominance, fragile health systems and the proliferation of vertical health programmes all present a risk to optimum health care services in the Arab world [21].

Our study shows that strong cultural taboos in the Arab world play a huge role in undermining discussions of SRH, which, in turn, means that a lack of knowledge has created public health problems for women and society at large. Cultural taboos seem to perpetuate a sense of fear of stigma around SRH problems. Similarly, Aldosari (2017) study about public health issues in Saudi Arabia revealed several similarities between Egypt and Saudi Arabia. For example, the Saudi health system is gendered which influences women’s health through treating women as objects rather than agents. Although the regulations restrict women’s capacity to seek medical support for unintended or unwanted pregnancy as they ask for their husband’s permission in the regulations of MOH for a lifesaving abortion, which indicates the impact of patriarchal norms on the Saudi health system [41].

In contrast, according to Bashour et al. (2015), Tunisia sponsored research on young people’s SRH from early 1990 and, in recent years, initiated health clinics for adolescents and unmarried woman in urban places. Tunisia also facilitated access to RH services in all health units, schools, and universities. It also ensured the need for training health care providers on active communication, education and providing quality services to young people. It has also worked on reinforcing collaboration and partnership with all the organizations and sectors working on youth issues [21].

[42] Finally, women’s limited knowledge of the various types of SRH and the advantages and disadvantages of contraceptive methods highlights the need to emphasize educational programmes and tools, and media awareness campaigns to equip women with information and knowledge about SRH services and information. This could help females from adolescence to adulthood to make informed decisions about their SRH and improve their utilization of these services. Women in this study exhibited very good attitudes and responses about introducing SRH programmes.

## Conclusion

The study findings revealed that married women’s access to and utilization of SRH information and services was low in Ismailia, Egypt. Married women’s experiences of accessing SRH services and information were influenced by intersecting factors located at the micro and macro levels. These intersected factors (e.g., power dynamics, socioeconomic factors, cultural norms, and religious misconception) shaped oppression and privilege structures which created unequal access to SRH information and services. Moreover, the study found that most of married women in this study had limited knowledge and information about their SRH and rights, and about the advantages and disadvantages of the family planning methods. The macro level factors included gender roles, parental power relations, and patriarchy which intersected with cultural and religious norms. These intersected factors played a vital role in negatively affecting women’s access to SRH service and information. In addition, the women in our study could have benefited from accessing SRH information at a younger age. Lessons could be learned from experiences in Arab countries like Tunisia where sexual and reproductive health education and information was introduced to young women in schools, universities, and health clinics. Through understanding the intersectional factors, meaningful interventions could be adopted to provide and enhance access to SRH services and information as well as address SRH rights and needs. There is an urgent need to empower women before and after marriage with accurate, safe, and affordable SRH services and information that could have life-long benefits to protect them. There is a necessity for building quality parental relationships for women before and after marriage in order to promote positive SRH attitudes and behaviour. There is a demand to emphasize educational programmes, media awareness campaigns, and educational tools to equip women with information and knowledge about SRH services and information. This could help females from adolescence to adulthood to make informed decisions about their SRH and improve their utilization of the services. Further research, both qualitative and quantitative, is needed to better understand the views of multisectoral stakeholders including the government, and public and private service providers on the barriers and opportunities for SRH services in Egypt.

## Data Availability

The datasets used and/or analyzed in relation to the current study are available from the corresponding author upon reasonable request.
